# Supplementary material for: Analysis of PPARγ Signaling Activity in Psoriasis
Source: Int J Mol Sci. 2021 Aug 10;22(16):8603. doi: 10.3390/ijms22168603 (PMC8395241; doi:10.3390/ijms22168603)
Supplement: Supplementary file 1 [file ijms-22-08603-s001.zip › Supplemental materials_Analysis of PPARg signaling activity in psoriasis/Pathway models/Models images and html files/PPARG inhibits phenotypes assotiated with psoriasis/143116.html]

oxidative stress


# Cell Process oxidative stress

|  |  |
| --- | --- |
| URN | urn:agi-cellproc:oxidative%20stress |
| Total Entities | 1 |
| Connectivity | 12887 |
| Name | oxidative stress |

---

|  |  |
| --- | --- |
| ChildConcepts | ROS generation |

---

|  |  |
| --- | --- |
| Pathway | Longevity Related Drugs |
|  | Oxidative Stress in Amyotrophic Lateral Sclerosis |
|  | Alzheimer's Disease |
|  | Skin Aging |
|  | High Level of Homocystine Effects (Methylation Cycle) |
|  | Melatonin Effects on Circadian Cycle |
|  | Autophagic-Lysosomal System Decline Associated with Aging |
|  | Fatty Acids Cause Oxidative Stress and Mitochondria Decline in Aging |
|  | Protein Oxidation and Aggregation in Aging |
|  | Necrosis |
|  | Positive Acute Phase Proteins Synthesis |
|  | Melatonin in Cell Survival and Antioxidant Response |
|  | Reperfusion Injury in Myocardial ischemia |
|  | Renin-Angiotensin-Aldosterone System in Myocardial ischemia |
|  | NO Synthesis in Early Vasospasm |
|  | Apoptotic Keratinocytes Clearance Recession in Systemic Lupus Erythematosus |
|  | Vitiligo |
|  | Humoral Immunity in Vitiligo |
|  | Macrophage Function in Diabetes Mellitus Type 1 |
|  | beta-Cell Death in Diabetes Mellitus Type 2 |
|  | Polyol Pathway in Diabetic Microangiopathy |
|  | Peripheral Tissue Microangiopathy |
|  | Tubular Cell Dysfunction in Progressive Diabetic Nephropathy |
|  | Oxidative Stress in Diabetic Neuropathy |
|  | AGE Related Insulin Resistance in Skeletal Muscles |
|  | Lipid Metabolism Impairement in non-Alcoholic Fatty Liver Disease |
|  | beta-Cell Recovery in Diabetes Mellitus |
|  | Hashimoto's Thyroiditis Triggers |
|  | Macrophage Dysfunction |
|  | Genes with Susceptibility for Crohn's Disease and Ulcerative Colitis |
|  | SERPINA1 Associated Pulmonary Emphysema |
|  | Club-Cell Role in Asthma |
|  | CFTR Facilitates Oxidative Stress in Airway Epithelium in CF |
|  | Airway Epithelial Cell Dysfunction |
|  | PPARGC1A Repression in Huntington Disease |
|  | Glutamate Toxiicity Effects on Striatum Medium Spiny Neurons in Hantington's Disease |
|  | Oxytocin Signaling in Brain Nerve Cell (Hypothesis) |
|  | Gluten Impact on Neuronal System (Hypothesis) |
|  | beta-Casomorphins/Opioid Receptors Affect Brain Function (Hypothesis) |
|  | Ca2+ Overload in Muscular Dystrophies |
|  | Muscular Dystrophies |
|  | Dystrophin Glycoprotein Complex Signaling in Muscular Dystrophies |
|  | Muscular Dystrophy, Facioscapulohumeral |
|  | Mitochondrial Dysfunction in Alzheimer's Disease |
|  | Estrogen Effects in Mitochondria in Alzheimer Disease |
|  | APP and Glutamate Signaling Cause Neuronal Dysfunction in Alzheimer Disease |
|  | Mitochondrial Respiratory Chain Dysfunction |
|  | APP Processing in Alzheimer Disease |
|  | Tricarboxylic Acid Cycle Enzymes Involvement in Alzheimer Disease |
|  | Metals and Amyloid beta Toxicity in Alzheimer's Disease |
|  | Ca2+ Toxicity in Neurons |
|  | PARK7 and LRRK2 in Young Onset Parkinson's Disease |
|  | Neurotoxin Effects in Parkinson's Disease |
|  | SNCA in Young Onset Parkinson's Disease |
|  | SOD1 Mutation |
|  | Glutamate Mediated Excitotoxicity in Amyotrophic Lateral Sclerosis |
|  | FOXL2 Mutation in Ovarian Dysgenesis |
|  | Oxidative Stress, All-Trans-Retinal and Lipofuscin Toxicity in AMD |
|  | TLR3 Role in Retinal Pigment Epithelial Cell |
|  | Alveolar Epithelial Cell Dysfunction in COPD |
|  | Podocyte Dysfunction in Glomerulonephritis |
|  | Hyperammonemia Type I |
|  | Citrullinemia Type I |
|  | Argininemia |
|  | Ammonia Effects on Brain Cells |
|  | Citrullinemia Type II |
|  | Argininosuccinic Aciduria in Urea Cycle Disorders |
|  | Hyperammonemia Type II |
|  | Cyclosporine Induced Nephrotoxicity |
|  | Glutamine in Cancer Metabolism |
|  | Mutations in Krebs Cycle Enzymes in Cancer |
|  | Tumor Infiltrating Macrophages in Cancer Progression and Immune Escape |
|  | EZH2 Methyltransferase Role in Cancer |
|  | FOXO1 Signaling |
|  | Glutamate Mediated Neurotoxicity |
|  | Oxidative Stress Protection via AHR-NFE2L2-NQO1 Signaling in Keratinocytes |
|  | New Pathway (1) |
|  | PPARG inhibits phenotypes assotiated with psoriasis |
|  | efects selected |
|  | 2\_Podocyte dysfunction in glomerulonephritis |
|  | 2\_Oxidative stress, all-trans-retinal, and lipofuscin toxicity in agerelated macular degeneration |
|  | 1\_Cigarette smoke causes dysfunction and production of inflammatory mediators in alveolar epithelial cells |
|  | 2\_Hyperglycemia and hyperlipidemia trigger beta-cell apoptosis |
|  | 3\_2\_Beta-cell proliferation and compensation: neogenesis/dedifferentiation |
|  | 2\_Ion channel CFTR failure in cystic fibrosis airway epithelium (class III-IV mutations) |
|  | 1\_Microangiopathy in diabetic neuropathy |
|  | 1\_Glutamate and calcium excitotoxicity of motor neurons in ALS |
|  | 2\_Mutations in SOD1 and associated proteins cause motor neuron death in familial ALS |
|  | 1\_ab\_Amyloid-beta in Alzheimer's disease |

---

|  |  |
| --- | --- |
| MedScan ID | 4003443 |

---

|  |  |
| --- | --- |
| Alias | oxidative damages |
|  | oxygen radical damages |
|  | free radical-induced damage |
|  | attack of hydroxyl radical |
|  | free radical-induced damages |
|  | oxidative stress |
|  | oxygen-free radical damages |
|  | free radical damage |
|  | hydroxyl radical attack |
|  | hydroxyl radical attacks |
|  | oxidant stresses |
|  | site-specific oxygen radical damage |
|  | oxidative injury |
|  | site-specific hydroxyl radical damage |
|  | oxygen radical damage |
|  | oxygen free radical-induced oxidative stress |
|  | Oxidative Stresses |
|  | oxidative injuries |
|  | free radical damages |
|  | oxidative damaging |
|  | oxidative stressing |
|  | oxygen-free radical damage |
|  | oxidative damage |

---

|  |  |
| --- | --- |
| MeSH ID | D018384 |
|  | M0027610 |

---
